# White Matter Microstructural Changes Following Quadrato Motor Training: A Longitudinal Study

**DOI:** 10.3389/fnhum.2017.00590

**Published:** 2017-12-07

**Authors:** Claudia Piervincenzi, Tal D. Ben-Soussan, Federica Mauro, Carlo A. Mallio, Yuri Errante, Carlo C. Quattrocchi, Filippo Carducci

**Affiliations:** ^1^Neuroimaging Laboratory, Department of Physiology and Pharmacology, Sapienza University of Rome, Rome, Italy; ^2^Research Institute for Neuroscience, Education and Didactics, Patrizio Paoletti Foundation, Assisi, Italy; ^3^Departmental Faculty of Medicine and Surgery, Università Campus Bio-Medico di Roma, Rome, Italy

**Keywords:** Quadrato Motor Training, diffusion tensor imaging, mindfulness, creativity, general self-efficacy

## Abstract

Diffusion tensor imaging (DTI) is an important way to characterize white matter (WM) microstructural changes. While several cross-sectional DTI studies investigated possible links between mindfulness practices and WM, only few longitudinal investigations focused on the effects of these practices on WM architecture, behavioral change, and the relationship between them. To this aim, in the current study, we chose to conduct an unbiased tract-based spatial statistics (TBSS) analysis (*n* = 35 healthy participants) to identify longitudinal changes in WM diffusion parameters following 6 and 12 weeks of daily Quadrato Motor Training (QMT), a whole-body mindful movement practice aimed at improving well-being by enhancing attention, coordination, and creativity. We also investigated the possible relationship between training-induced WM changes and concomitant changes in creativity, self-efficacy, and motivation. Our results indicate that following 6 weeks of daily QMT, there was a bilateral increase of fractional anisotropy (FA) in tracts related to sensorimotor and cognitive functions, including the corticospinal tracts, anterior thalamic radiations, and uncinate fasciculi, as well as in the left inferior fronto-occipital, superior and inferior longitudinal fasciculi. Interestingly, significant FA increments were still present after 12 weeks of QMT in most of the above WM tracts, but only in the left hemisphere. FA increase was accompanied by a significant decrease of radial diffusivity (RD), supporting the leading role of myelination processes in training-related FA changes. Finally, significant correlations were found between training-induced diffusion changes and increased self-efficacy as well as creativity. Together, these findings suggest that QMT can improve WM integrity and support the existence of possible relationships between training-related WM microstructural changes and behavioral change.

## Introduction

In the last two decades, white matter (WM) microstructural changes of the human brain have been widely described *in vivo* using the diffusion tensor imaging (DTI) magnetic resonance technique (Basser et al., 1994; Pierpaoli et al., 1996). DTI is sensitive to the magnitude and orientation of water diffusion throughout brain tissue, and exploits this information to calculate several diffusion parameters through a tensor model. The most commonly used DTI parameter is the fractional anisotropy (FA), which represents the degree of directionality of water diffusivity. Higher FA values are thought to reflect better WM integrity as a result of greater intravoxel coherence of fiber orientation, axon density and diameter and/or myelination (Beaulieu et al., 1996; Sen and Basser, 2005; Caminiti et al., 2013). Instead, reduced FA values have been found in aging and in psychiatric and neurological disorders (Taubert et al., 2012; Barysheva et al., 2013; Mayo et al., 2017).

Several DTI studies which have aimed at characterizing the mechanisms of FA change and at determining if these changes are the result of axon morphological modification or myelination, have also examined other diffusion parameters, such as axial and radial diffusivity (AD, RD), in the location where FA significantly changes, respectively (Wheeler-Kingshott and Cercignani, 2009; Bennett et al., 2010; Tang et al., 2012). As a matter of fact, alterations in AD have been associated with changes in axon morphology (Kumar et al., 2012), and lower AD values have been generally related to decrements of axonal density or caliber (Mac Donald et al., 2007). On the other hand, RD has been generally associated with myelination (Song et al., 2002, 2005), where RD decrease has been thought to reflect increased myelination (Keller and Just, 2009; Bennett et al., 2010).

Neuroimaging studies have consistently demonstrated that training and learning can modify the WM microstructure of the brain, determining related changes in behavior and/or performance (Taubert et al., 2012; Zatorre et al., 2012). WM microstructural changes have been reported by several longitudinal DTI studies after various amounts of training in different domains, such as visuo-motor coordination and whole-body balancing tasks (Scholz et al., 2009; Taubert et al., 2010), musical (Imfeld et al., 2009; Steele et al., 2013), working memory (Takeuchi et al., 2010; Salminen et al., 2016) and reasoning trainings (Mackey et al., 2012).

Mindfulness practices have also been found to induce WM microstructural changes (for recent reviews see Fox et al., 2014; Tang et al., 2015). Mindfulness has been defined as “the awareness that emerges through paying attention, on purpose, in the present moment, and non-judgmentally to the unfolding of experience moment by moment” (Kabat-Zinn, 2003). Mindfulness practices were reported to improve psychological health and well-being (Keng et al., 2011), as well as cognitive functions, such as attention, memory, and concentration (Jha et al., 2007; MacLean et al., 2010; Mrazek et al., 2013). In particular, mindful movement practices, such as mindful walking, Yoga, Tai Chi and Aikido involve intentional movement while bringing awareness to the body and its location in space (Kabat-Zinn, 2013).

Many cross-sectional studies have demonstrated microstructural WM differences between mindfulness practitioners and controls, as well as between novice and expert practitioners (Luders et al., 2011; Kang et al., 2013). However, only few studies have performed longitudinal investigations to establish the effects of mindfulness practices on WM architecture and assess possible relationships between WM changes and concomitant changes in behavior. Of note, these studies only focused on the longitudinal effect of *sitting* mindfulness practices, with subjects tested only two times (pre-post training) and with some important limitations, related to possible cultural-genetic differences of the samples used (Tang et al., 2012) or relatively small sample size (Holzel et al., 2016). Furthermore, these studies used standard approaches for the analysis of longitudinal diffusion data, while recent developments have highlighted the importance of processing each subject’s data at multiple time points in an unbiased way, especially reporting inaccuracies of longitudinal measures obtained with standard registration methods (Yushkevich et al., 2010; Reuter et al., 2012; Keihaninejad et al., 2013).

To our knowledge, no longitudinal investigations examined the effects of whole-body mindful movement practices on WM architecture and the possible relation between WM and behavioral changes. In addition, none of the previous studies investigated if there is a ceiling beyond which further mindful training results in no further structural changes (Fox et al., 2014).

Recently, a new whole-body mindful movement paradigm, the Quadrato Motor Training (QMT), was developed to enhance attention, coordination, creativity, and mindfulness (Dotan Ben-Soussan et al., 2013; Ben-Soussan et al., 2014b). The QMT requires standing at one corner of a square and making movements toward different corners in response to verbal instructions (see *The Quadrato Motor Training* paragraph in the Methods for a detailed description of the training). The QMT requires a state of enhanced attention to the motor response and cognitive processing for producing the correct direction of movement to the next corner in the Quadrato space (Dotan Ben-Soussan et al., 2013).

Quadrato Motor Training incorporates all the three interdependent phases of a mindful act (Kabat-Zinn, 2013): (1) suspension from the habitual act of allowing the mind and body to go where they want, (2) redirection of attention (toward the external cue and the internally generated movement), and (3) receptivity toward the experience (the subject stands in a receptive manner in between instructions, without correcting motor or decision errors) (Depraz et al., 2000). Importantly, respect to other mindful movement practices such as Tai Chi and Aikido, QMT has the advantage of being a relatively short training, very easy to perform and practice in limited spaces.

In the last years, QMT has been deeply investigated in order to highlight eventual behavioral and neurophysiological changes induced by this whole-body mindful training.

At the behavioral level, it has been demonstrated that a session or a month of daily QMT improves reaction times, ideational flexibility and spatial cognition, in contrast to several control groups, such as simple motor training and verbal training (Dotan Ben-Soussan et al., 2013; Ben-Soussan et al., 2015a). QMT also proved its validity in improving emotional well-being following a month of daily training, compared to breathing meditation as well as a simple motor training (Ben-Soussan, 2014). Creativity and general self-efficacy are important aspects of psychological well-being, previously related to mindfulness practices (Capurso et al., 2014; Charoensukmongkol, 2014; Sanaei et al., 2014; Tabak et al., 2015; Mehdizadeh Zare Anari and Shafiei, 2016).

At the electrophysiological level, previous studies showed that a session of QMT practice significantly increases inter- and intra-hemispheric EEG alpha (α; 8–12 Hz) coherence within frontal and parietal areas in healthy adults respect to controls (Dotan Ben-Soussan et al., 2013). Interestingly, these changes significantly correlated with creativity improvements in ideational flexibility, supporting the idea of a connection between functional connectivity in the α range and enhanced creativity (Ben-Soussan et al., 2015b). Furthermore, using magnetoencephalography (MEG), Ben-Soussan et al. (2014a) also found that a month of daily QMT increases cerebellar oscillatory α power and inter-hemispheric α coherence in dyslexics respect to normal readers which served as controls, also improving the reading performance of both groups.

As mentioned above, no previous longitudinal study looked at the effects of whole-body mindful movement practices on WM architecture and their relationship with concomitant behavioral changes. Therefore, the first aim of the present study was to investigate the longitudinal effects of QMT on WM microstructure. In this way, we also aimed at increasing our understanding of the possible effects of this practice at a neuroanatomical level.

The second aim was to identify possible relationships between training-related longitudinal WM changes and concomitant changes in creativity, general self-efficacy and motivation. Of note, respect to previous conventional longitudinal studies in which the subjects were tested only two times, the participants in the current study were tested three times over a period of 12 weeks of daily QMT, to explore the trend of training-related microstructural WM changes over time. Furthermore, in the current work a longitudinal analysis of diffusion data was carried out following an unbiased tensor-based registration approach (Keihaninejad et al., 2013), which provides more accurate and sensitive longitudinal measures respect to standard FA-based image registration methods (Jones et al., 2002; Smith et al., 2006), followed by a whole brain tract-based spatial statistics (TBSS) analysis.

## Materials and Methods

The present study is part of a larger project aimed at investigating the longitudinal effect of QMT using different brain imaging techniques. For this reason, the experimental procedure also includes electrophysiological measures, which have been analyzed and discussed elsewhere (Lasaponara et al., 2017).

### Subjects

We recruited 50 healthy volunteers. Following the inclusion and exclusion criteria reported in **Table 1**, 4 subjects were excluded due to the presence of WM lesion, 3 subjects because they were left-handed, 6 subjects because of MRI incomplete protocol and 2 subjects because of lack in complying motor exercise. Thus, the analyses were conducted in a group of 35 healthy right-handed subjects (21 women and 14 men, mean age ± SD: 35 ± 5 and 36 ± 5 years, respectively).

**Table 1:** Inclusion and exclusion criteria of the present study.

**Inclusion criteria**
– Age between 25 and 45 year
– Right-handedness
– No history of current or past drug addiction/abuse or antidepressant use
– No motor, emotional, cognitive or developmental coordination disorders
– No previous practice of the QMT or other motor activation programs
**Exclusion criteria**
– History of traumatic injury, previous neurosurgery, stroke, inflammatory/infective brain disease
– Co-morbidity of congenital metabolic diseases or malformations
– Diagnosis of one histologically proven primary cancer (< 1 years)
– Vitamin B12 deficiency, positive serology for secondary dementia (RPR/ VDRL, HIV, anti-Borrelia), abnormal thyroid function
– Clinical evidence of depression or other psychiatric conditions, epilepsy, drugs or alcohol addiction (according to DSM IV-TR)
– Severe cognitive impairment (Mini Mental State Examination ≤ 24)
– Diagnosis of malnutrition
– Chronic or acute inflammatory disease
– Hearing or visual impairment or motor deficits incompatible with the workout
– Hormone replacement therapy
– Current or recent history of smoking (i.e., not smoking during the last year)

### Ethics Statement

All procedures were explained to participants, verifying sufficient understanding and written informed consent was obtained in accordance with the declaration of Helsinki. The ethical committee of the Università Campus Bio-Medico di Roma, Rome, Italy, approved the experimental phase I study entitled “Effect of quadrato MOtor Training On the BRAIN of healthy volunteers” (MOTO-BRAIN, 09/14 PAR ComEt CBM. Compliance with GCP (Good Clinical Practice) was warranted and data were collected following the ALCOA (Attributable, Legible, Contemporaneous, Original and Accurate) algorithm. The TREND checklist was also accomplished (S1 TREND Checklist). Participants were free to interrupt the QMT and drop-out from the study at any time for any reason, without any prejudice.

### Procedure

**FIGURE 1:**
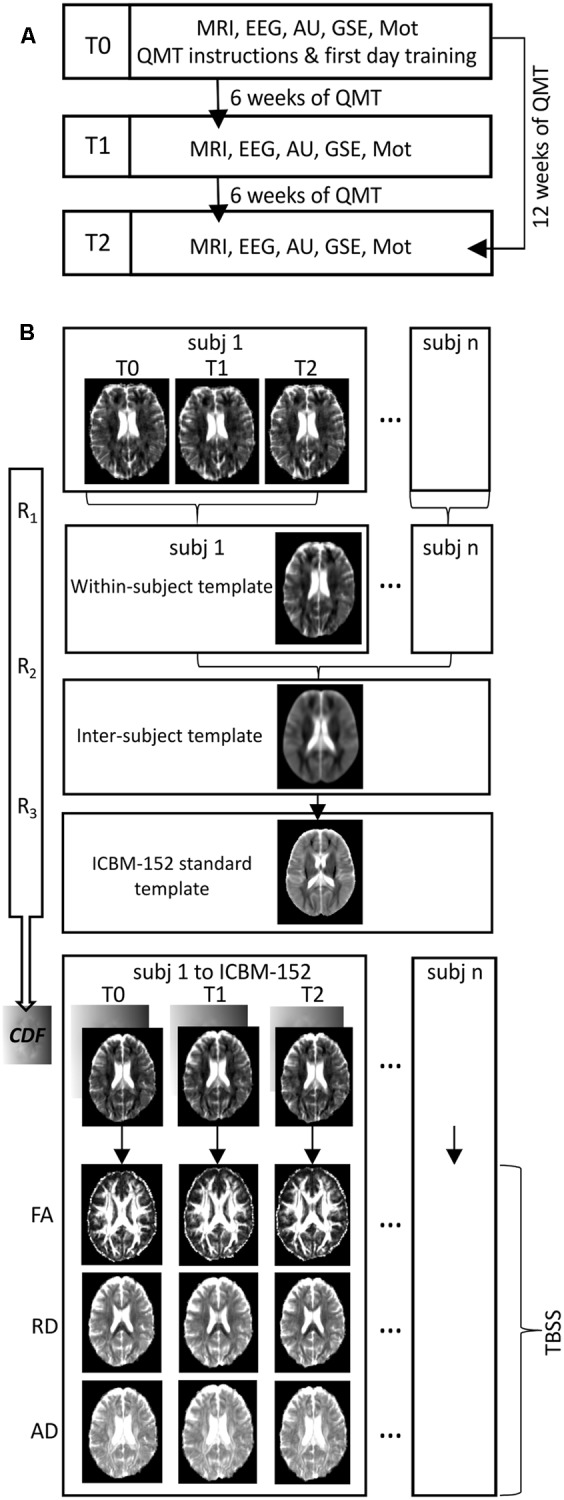
**(A)** Flowchart of the experiment. A schematic representation of the longitudinal organization of the experimental procedure over time (MRI, Magnetic Resonance Imaging scanning; EEG, Electroencephalographic recordings; AU, Alternative Uses task; GSE, General Self Efficacy scale; Mot, Motivation scale). **(B)** Flowchart of processing pipeline for longitudinal diffusion tensor imaging analysis (R_1_, R_2_, R_3_: iterative processes of rigid, affine and non-linear diffeomorphic registrations; CDF: combined deformation field that define the mapping from subject-space to within-subject template, within-subject template to inter-subject template and inter-subject template to ICBM-152 standard template; FA, fractional anisotropy; RD, radial diffusivity; AD, axial diffusivity; TBSS, Tract-based Spatial Statistics).

Volunteers were asked to consent to a longitudinal evaluation at our institution as a pre-requisite for recruitment. The longitudinal protocol consisted of three time points: (i) baseline – the day of recruitment (T0), (ii) 6 weeks after daily QMT (T1), and (iii) 12 weeks after daily QMT (T2) (see **Figure 1A** for an overview of the experimental protocol). We chose these time-intervals since they have been both widely used in previous longitudinal studies focusing on structural training-related changes (Draganski et al., 2004; Scholz et al., 2009; Taubert et al., 2010; Mackey et al., 2012).

To check for compliance to the exercise, subjects were asked to fill up a personal diary on a daily basis and collect information about their practice and habits during the period of exercise. At each time point, the diary needed to be accurate and complete as a pre-requisite for proceeding to the next time point measurements.

At each time point, participants underwent magnetic resonance imaging (MRI) scanning brain and electroencephalography (EEG). Clinical interview and cognitive examination were performed in a dedicated room beside the MRI magnet site. Handedness was assessed by the Edinburgh Handedness Inventory (Oldfield, 1971). Creativity was assessed using the Alternate Uses (AU) Task (Guilford, 1978; Ben-Soussan et al., 2015a). General Self-Efficacy (GSE) test (Chen and Gully, 1997) and Motivation (Mot) scale (Pintrich, 1991) were also administered to investigate perceived self-efficacy and motivation, respectively.

#### The Quadrato Motor Training

**FIGURE 2:**
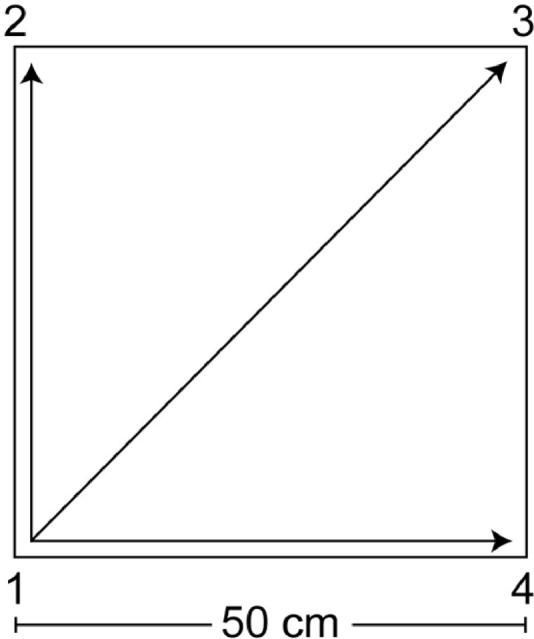
Graphical illustration of the Quadrato Motor Training. The participants stood in a quiet room at one corner of a 0.5 m × 0.5 m square and made movements to the different corners of the square in response to verbal instructions given by an audio tape recording, indicating the next corner to which the participant should move (for example, “one four” means move from corner 1 to corner 4). Participants were instructed to keep their eyes focused straight ahead, their hands loose at the side of the body and to begin all movements with the leg closest to the center of the square. In this longitudinal experimental protocol, the daily training consisted of a sequence of 69 commands lasting 7 min.

The QMT, created by Patrizio Paoletti, requires standing at one corner of 0.5 m × 0.5 m square and making movements to different corners of the square in response to verbal instructions given by an audio tape recording indicating the next corner to which the participant should move (see **Figure 2**). In the QMT, there are 3 optional directions of movement, and the movement is always in one step. Each movement can be forward, backward, left, right, or diagonal, thus the training consists of 12 possible movements (3 directions × 4 corners): 2 forward, 2 backward, 2 left, 2 right and 4 diagonals. The instructions given to the participants were to (i) keep eyes focused straight ahead and hands loose at the side of the body, (ii) immediately continue with the next instruction and (iii) do not stop in case of mistakes. Daily training consisted of a sequence of 69 commands lasting a total of 7 min, with a movement sequence paced at a rate of an average of 0.5 Hz (comparable to a slow walking rate). The participants were also instructed to begin all movements with the leg closest to the center of the square.

#### Alternate Uses (AU) Task

The AUs Task is an established psychometric test to assess divergent creative thinking (Guilford, 1968, 1978), previously used to study changes in creativity following whole-body training (Netz et al., 2007; Fink et al., 2009; Colzato et al., 2012) including the QMT (Dotan Ben-Soussan et al., 2013; Ben-Soussan et al., 2015a; Venditti et al., 2015). Sustaining and improving creativity may serve a significant role in maintaining cognitive and emotional well-being and health (Schmid, 2005). In this task, the participant is required to name as many different ways as possible in which a given item might be used within a 1-min time frame. Three basic measures were computed from the AU task: *ideational fluency*, defined as the total number of generated responses, *ideational flexibility*, defined as the tendency to generate a heterogeneous pool of responses, and *originality*, defined as the capacity to provide unusual or unique responses.

#### General Self Efficacy (GSE) Scale

The GSE (Chen and Gully, 1997) is a 14-item psychometric scale designed to assess a general sense of perceived self-efficacy, defined as a person’s belief in his/her overall ability to perform well across a variety of difficult demands in life (Chen et al., 2001). Physical exercise as well as different mindfulness practices have been previously found to enhance self-efficacy (McAuley and Blissmer, 2000; Chang et al., 2010; Charoensukmongkol, 2014; Sanaei et al., 2014; Paoletti et al., 2017). High general self-efficacy is considered a resource that buffers against stressful experiences, as high self-efficacious individuals perceive demands as challenging, not as threatening (Jerusalem and Schwarzer, 1992). In this task, the participant was asked to state the degree to which he or she agrees with each of the statements in the questionnaire on a scale ranging from “1” (strongly disagree) to “5” (strongly agree). The total scores on the questionnaire range from 14 to 70, with the highest score reflecting higher self-efficacy.

#### The Motivation (Mot) Scale

The Mot scale was inspired by the Motivated Strategies for Learning Questionnaire (MSLQ), (Pintrich, 1991). The Mot is a 10-item scale designed to assess a sense of perceived motivation and enjoyment. Motivation involves the mental process people use to activate, sustain, and maintain behavior (Pintrich and Maehr, 2002). It has been demonstrated that confident and motivated people tend to try hard, persist, and perform better than those who doubt of their capabilities (Schunk, 1991; Pintrich and Schrauben, 1992; Pintrich, 1999).

In this task, the participant was asked to state the degree to which he or she agrees with each of the statements in the questionnaire on a scale ranging from “1” (strongly disagree) to “7” (strongly agree) related to the level of motivation and enjoyment prior, during and following the training. The total scores on the questionnaire range from 10 to 70, with the highest score reflecting higher motivation.

### MRI Data Acquisition

Imaging data were acquired using a Siemens 1.5-T MAGNETOM Avanto (Siemens, Erlangen, Germany) whole body scanner equipped with a 12-element designed Head Matrix coil, as part of the standard system configuration. Diffusion weighted images (DWIs) were acquired using an axial pulsed-gradient spin-echo echo-planar sequence (7600/103; 38 sections; section thickness, 3.0 mm with no intersection gap), with diffusion-encoding gradients applied in 12 non-collinear directions (b factor 0 and 1000 s/mm^2^; number of acquired signals, four). A 2D fluid attenuated inversion recovery (FLAIR) T2 weighted scan was also used to exclude the presence of small vessel ischemic disease and other supra- or infra-tentorial brain lesions (TR = 11460 ms, TE = 102 ms, TI = 2360 ms, FOV = 280 mm × 330 mm, NEX = 2, matrix = 248 × 320, 1.00 × 1.00 mm^2^ in-plane resolution, horizontal slices with slice thickness of 3.0 mm and no gap). Structural images were collected using a sagittal magnetization-prepared rapid acquisition gradient echo (MPRAGE) T1-weighted sequence (TR = 2400 ms, TE = 3.61 ms, TI = 1000 ms, flip angle = 15°, FOV = 240 mm × 280 mm, NEX = 1, matrix = 192 × 192, 1.00 × 1.00 mm^2^ in-plane resolution, horizontal slices with slice thickness of 1.2 mm and no gap). Whole brain functional scans were also acquired in 25 contiguous axial slices approximately parallel to the anterior-posterior commissure plane with interleaved multi-slice T2 echo-planar imaging (TR = 3560 ms, TE = 50 ms, field of view = 22 cm, flip angle = 90°, voxel size = 3.4 × 3.4 × 3 mm, slice thickness = 3 mm, no inter-slice gap, 135 volumes). Since the present paper focused on WM microstructural QMT- related changes, resting-state data will not be discussed further.

### MRI Data Analysis

To avoid a type I error induced by the effect of WM hyperintensities on brain connectivity results, two expert radiologists (CCQ, YE) examined all MRIs. Subjects were excluded when more than 3 lesions with a maximum diameter of 5 mm were detected in the subcortical or periventricular WM on axial FLAIR images (Quattrocchi et al., 2015).

#### Preprocessing of Diffusion Data

All DWIs were visually inspected for artifacts and preprocessed using different tools from FDT (FMRIB Diffusion Toolbox, part of FSL (FMRIB’s Software Library v.5.0.8,^1^; Smith et al., 2004). Images were corrected for eddy current distortion and head motion using a 12 parameter affine registration to the first no-diffusion weighted volume of each subject, and the gradient directions were rotated accordingly (Leemans and Jones, 2009). Corrected images were skull-stripped using BET (Smith, 2002). Diffusion tensor images were then generated for each participant and each time point using the Diffusion Tensor Imaging ToolKit software package (DTI-TK^2^) (Zhang et al., 2006). An unbiased longitudinal analysis approach was chosen for the registration of DTI data (Keihaninejad et al., 2013) using DTI-TK, which applies a registration algorithm that leverages the full diffusion tensor information (rather than scalar features) to drive the registration and improve the alignment of WM structures (Wang et al., 2011) (see **Figure 1B** for an overview of analysis pipeline). The tensor-based registration method using DTI-TK showed a good reproducibility of DTI metrics when performing repeated DTI measurements (Keihaninejad et al., 2013). For each participant, a within-subject template was generated from the three time points tensor images using an iterative process of rigid, affine and non-linear diffeomorphic registrations. The within-subject templates were then used to create a study-specific inter-subject template using the same iterative process of linear and non-linear registrations (Keihaninejad et al., 2013). The inter-subject template was subsequently registered to an ICBM-152 space enhanced diffusion template (Zhang et al., 2011), again using the same sequence of registrations. Finally, we computed for each subject the combined deformation field that define the mapping from subject-space to within-subject template, within-subject template to inter-subject template and inter-subject template to ICBM-152 standard template. These combined fields were then used to normalize each corresponding subject’s DTI data to the ICBM-152 template. For each participant and each time point FA, RD and AD maps were generated using the normalized tensor images.

#### Tract-Based Spatial Statistic (TBSS) Analysis of DTI Data

FA, RD, and AD data from each participant were furtherly analyzed using the Tract-Based Spatial Statistics (TBSS; Smith et al., 2006) toolbox, available in FSL. The mean FA image was created and thinned to create a mean FA skeleton, which represents the centers of all tracts common to the group. Each participant’s FA image was then projected onto this common skeleton to minimize any residual misalignment of tracts. The skeleton projection was then applied to RD and AD images to create a separate skeleton representing the RD and AD values. Individual difference images between time points (T1-T0, T2-T0 and T2-T1) were finally obtained for FA, RD, and AD data.

### Statistical Analyses

To investigate on the effects of QMT on creativity, perceived self-efficacy and motivation, repeated measure analyses of variance (ANOVA) over the three time points (T0, T1, and T2) were performed, separately for ideational fluency, flexibility and originality AUs’ subscales scores, GSE and Mot measures. The differences between time points were finally computed for the all the AUs’ subscales scores, the GSE scores and the Mot scores. Statistical analyses on behavioral data were performed using Statistica v.7 software (StatSoft Inc., United States).

White matter microstructural changes were also investigated performing three separate one-sample *t*-tests on the difference images of FA, RD and AD, using age and gender as nuisance variables. RD and AD changes were only investigated within the regions where FA changes were found, to determine whether the FA changes are related to axon morphology (i.e., AD) or character of myelin (i.e., RD) (Wheeler-Kingshott and Cercignani, 2009; Tang et al., 2012; Taubert et al., 2012).

Voxelwise statistical analyses were carried out using permutation-based non-parametric statistics using the FSL Randomize permutation-based program (Nichols and Holmes, 2002) with 5,000 permutations. The statistical threshold was established with a family wise error corrected *p*-value (pFWE) < 0.05 with multiple comparison correction using threshold-free cluster enhancement (TFCE) (Smith and Nichols, 2009). Mean FA values were also extracted from each individual’s FA skeleton map and a repeated measures ANOVA was conducted on FA values at T0, T1, and T2, to explore the trend of FA changes in time.

Randomize tool (5,000 permutations) was also used to examine the statistical correlation between significant longitudinal changes of diffusion parameters and longitudinal behavioral changes. Resulting statistical maps were thresholded at pFWE < 0.05.

All the results were anatomically localized using the JHU ICBM-DTI-81 White-Matter Labels and the JHU White-Matter Tractography atlases included in the FSL distribution^3^.

## Results

### QMT-Related Behavioral Changes

As shown by repeated measures ANOVAs, QMT significantly increased the originality subscale score of the AU task over time (*p* < 0.05), but it does not have a significant effect on ideational fluency and ideational flexibility. Bonferroni *post hoc* tests were conducted on all the possible pairwise contrasts for the originality subscale scores, revealing that T1 scores were significantly higher than T0 scores (*p* < 0.05). GSE measures were also significantly increased over time and with the training (*p* < 0.005); Bonferroni *post hoc* test indicated that GSE scores at T2 were significantly higher than GSE scores at T0 and T1 (*p* < 0.005 and *p* < 0.05, respectively). Finally, the repeated measures ANOVA on Mot scores did not show a significant effect for time (*p* > 0.1).

### QMT-Related WM Microstructural Variations and Associated Behavioral Changes

**FIGURE 3:**
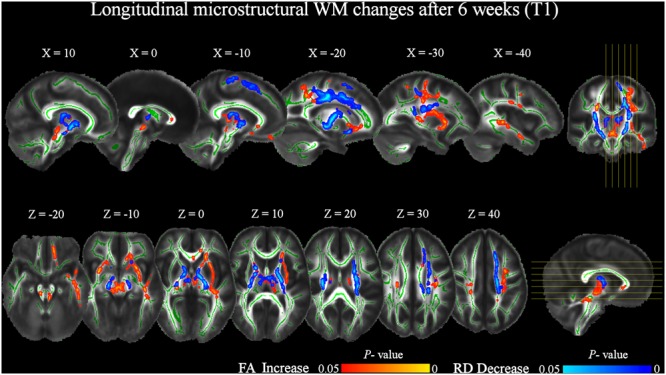
Significant increases in FA and decreases in RD after 6 weeks of daily QMT (T1) respect to T0 baseline (pFWE < 0.05, TFCE corrected). RD changes were investigated in the locations where FA significantly changed. The study-specific FA skeleton, representing the centers of principal WM tracts, is displayed in green, overlaid on the mean FA map. The vertical lines on the coronal view indicate the sagittal slices displayed. The horizontal lines on the sagittal view indicate the axial slices displayed. The red–yellow and blue–light blue color bars represent level of significance for FA increase and RD decrease, respectively.

At T1 respect to T0, TBSS analysis revealed a significant (pFWE < 0.05) FA increase of several WM tracts (**Figure 3** and **Table 2**), including corticospinal tracts, uncinate fasciculi, anterior thalamic radiations and internal capsules bilaterally, right external capsule, cerebral peduncle and left superior cerebellar peduncle. Increased FA was also found in the forceps minor at the level of the genu, and in the body of the corpus callosum. Finally, FA increments in the left hemisphere were found in the superior longitudinal fasciculus and its temporal part, inferior fronto-occipital and inferior longitudinal fasciculi.

**Table 2:** Significant increases in FA at T1 respect to T0 (pFWE < 0.05 TFCE-corrected).

			MNI coordinates			
Cluster size	*T*	*p*	*x*	*y*	*z*	WM structures
7409	5.93	<0.001	–16	–7	3	*Left posterior limb of internal capsule*
	5.79	<0.001	–20	–18	42	Left corticospinal tract
	4.32	0.007	–24	25	6	Left inferior fronto-occipital fasciculus, Left Uncinate fasciculus
	4.01	0.007	–34	4	2	Left superior longitudinal fasciculus
	3.57	0.008	–20	18	30	Left superior longitudinal fasciculus (temporal part)
	3.47	0.018	–3	21	–2	Forceps minor/*Genu of corpus callosum*
2266	4.93	0.003	–10	–17	16	Left anterior thalamic radiation
	3.69	0.019	9	–28	–11	Right anterior thalamic radiation
1163	4.44	0.021	10	–1	–4	Right anterior thalamic radiation
	3.89	0.023	28	–19	19	Right corticospinal tract
	2.77	0.038	18	–20	–7	Right corticospinal tract/*Right cerebral peduncle*
1159	4.86	0.019	–53	–37	–19	Left superior longitudinal fasciculus (temporal part)
	4.67	0.015	–37	–28	–2	Left inferior fronto-occipital fasciculus/*Left retrolenticular part of internal capsule*
	4.23	0.019	–42	–4	–26	Left inferior longitudinal fasciculus
316	3.74	0.021	–21	–53	60	Left superior longitudinal fasciculus
153	3.42	0.029	–46	–14	–11	Left inferior longitudinal fasciculus
128	4.05	0.043	17	7	8	Right anterior thalamic radiation/*Right anterior limb of internal capsule*
106	3.56	0.044	24	–18	35	Right corticospinal tract
75	2.79	0.046	33	5	–11	Right uncinate fasciculus/*Right external capsule*
45	3.13	0.047	–6	–39	–21	Left anterior thalamic radiation/*Left superior cerebellar peduncle*
44	2.55	0.047	17	19	–11	Right uncinate fasciculus/*Right external capsule*
28	2.23	0.047	–15	11	29	*Body of corpus callosum*
28	2.44	0.033	–33	–35	8	Left inferior longitudinal fasciculus/*Left retrolenticular part of internal capsule*

*Peak MNI coordinates (mm) within clusters were identified using a minimum peak-distance between local maxima of 20 mm. Only clusters showing a spatial extent of at least 20 contiguous voxels were reported. Anatomical localizations of peak MNI coordinates were established according to the JHU White-Matter Tractography and the JHU ICBM-DTI-81 White-Matter Labels (cursive font) atlases.*

The increase of FA at T1 respect to T0 was accompanied by a significant bilateral decrease of RD in corticospinal tracts and anterior thalamic radiations, also including the posterior limbs of internal capsule. RD decrements were also found in the left uncinate, inferior fronto-occipital, and superior longitudinal fasciculi, as well as in the right anterior limb of the internal capsule and cerebral peduncle (**Figure 3** and **Table 3**).

**Table 3:** Significant decreases in RD at T1 respect to T0 (pFWE < 0.05 TFCE-corrected).

			MNI coordinates			
Cluster size	*T*	*P*	*X*	*y*	*z*	WM structures
4615	4.82	<0.001	–16	–7	4	Left anterior thalamic radiation/*Left posterior limb of internal capsule*
	4.80	0.002	–12	–15	67	Left superior longitudinal fasciculus
	3.87	<0.001	–26	–20	22	Left corticospinal tract
	2.24	0.009	–23	21	5	Left inferior fronto-occipital fasciculus, Left uncinate fasciculus
906	5.27	0.007	–10	–16	16	Left anterior thalamic radiation
	3.68	0.011	10	–15	15	Right anterior thalamic radiation
468	4.42	0.012	12	–3	–4	Right anterior thalamic radiation
	2.31	0.035	24	–6	18	Right corticospinal tract/*Right anterior limb of internal capsule*
388	3.73	0.014	28	–19	18	Right corticospinal tract/*Right posterior limb of internal capsule*
122	3.47	0.032	18	–20	–7	Right corticospinal tract/*Right cerebral peduncle*
74	4.93	0.028	17	7	8	Right anterior thalamic radiation/*Right anterior limb of internal capsule*

*RD changes were investigated in the locations where FA significantly changed. Refer to **Table 2** for a detailed explanation of the table layout.*

**FIGURE 4:**
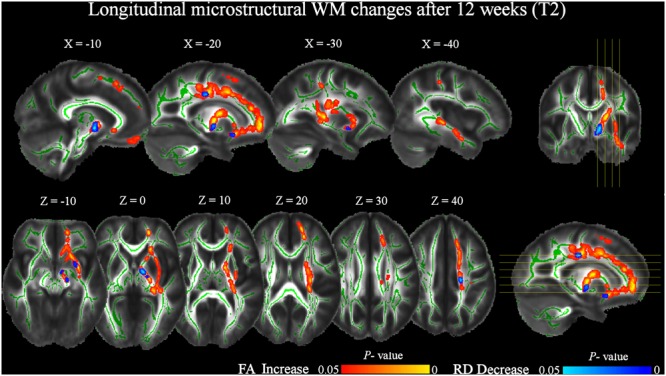
Significant increases in FA and decreases in RD after 12 weeks of daily QMT (T2) respect to T0 baseline (pFWE < 0.05, TFCE corrected). See **Figure 3** for additional details.

At T2 respect to T0, a significant (pFWE < 0.05) increase of FA was still present (**Figure 4** and **Table 4**). Notably, longitudinal changes were less widespread and only found in the left-hemisphere, including uncinate and inferior longitudinal fasciculi, forceps minor and corticospinal tract. Furthermore, FA increments were found in the anterior thalamic radiation and inferior fronto-occipital fasciculus. In this case, the increase of FA was accompanied by a significant decrease of RD only in the left anterior thalamic radiation and uncinate fasciculus (**Figure 4** and **Table 5**). No significant changes of FA and RD were found at T2 respect to T1. Furthermore, no significant changes of AD were found in the location where FA changed.

**Table 4:** Significant increases in FA at T2 respect to T0 (pFWE < 0.05 TFCE-corrected).

			MNI coordinates			
Cluster size	*T*	*p*	*X*	*y*	*Z*	WM structures
4337	4.89	0.012	–20	51	–2	Left anterior thalamic radiation
	4.79	0.016	–23	27	–2	Left uncinate fasciculus, Left inferior fronto-occipital fasciculus
	4.25	0.021	–16	53	18	Forceps minor
	4.05	0.014	–35	–3	–14	Left uncinate fasciculus
	3.90	0.023	–28	–28	7	Left inferior fronto-occipital fasciculus/*Left retrolenticular part of internal capsule*
	3.38	0.022	–22	–5	15	Left anterior thalamic radiation/*Left posterior limb of internal capsule*
	1.95	0.031	–43	–28	–7	Left inferior longitudinal fasciculus
653	4.57	0.025	–21	–24	40	Left corticospinal tract

*Refer to **Table 2** for a detailed explanation of the table layout.*

**Table 5:** Significant decreases in RD at T2 respect to T0 (pFWE < 0.05 TFCE-corrected), investigated in the locations where FA significantly changed.

			MNI coordinates			
Cluster size	*T*	*p*	*x*	*y*	*z*	WM structures
139	4.11	0.008	–10	–4	–6	Left anterior thalamic radiation
40	3.30	0.033	–27	10	–13	Left uncinate fasciculus

*Refer to **Table 2** for a detailed explanation of the table layout.*

**FIGURE 5:**
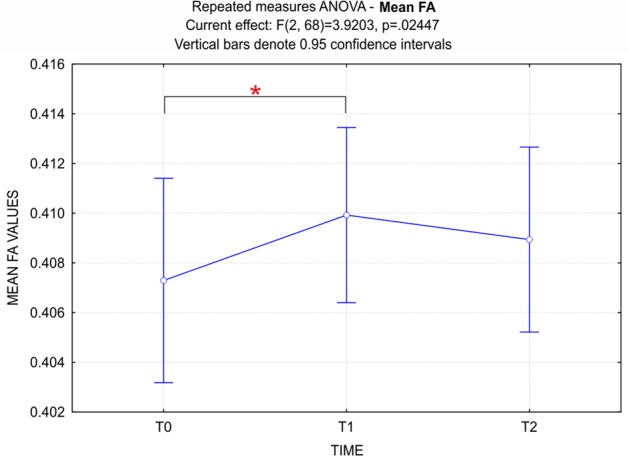
ANOVA repeated measures - Time (3) factor for mean FA values. Data show a significant principal effect for the Time factor. Bonferroni *post hoc* correction showed that FA values at T1 were significantly higher than at T0 (*p* < 0.05). It should be noted that, although not significantly different, FA values at T2 were lower than at T1.

The repeated measures ANOVA performed on the mean FA values at T0, T1, and T2, showed a significant effect for time (*p* < 0.05) (**Figure 5**). *Post hoc* comparisons using the Bonferroni test indicated that FA values at T1 were significantly higher than at T0 (*p* < 0.05). Interestingly, although not significantly different, FA values at T2 were lower than at T1.

The correlation analyses between significant longitudinal changes of diffusion parameters and behavioral changes yielded no FWE-corrected results. However, since previous studies already reported significant correlations between QMT-related changes of psychological well-being measures and electrophysiological indices (Dotan Ben-Soussan et al., 2013), proNGF levels (Venditti et al., 2015), and structural changes (Ben-Soussan et al., 2015a,c), the uncorrected statistical results (*p* < 0.005) are also reported in **Table 6**. Positive correlations were found between longitudinal increments of FA and both AUs originality and GSE scores at T1 respect to T0. More specifically, these correlations were similarly located in the right anterior thalamic radiation and left superior longitudinal fasciculus. In addition, the correlation between FA and GSE also included the left anterior thalamic radiation. Negative correlations were also found between longitudinal decrements of RD and improvements of both AUs originality and GSE scores at T1 respect to T0. The correlation between RD and originality was mainly located in the left superior longitudinal fasciculus, while the one between RD and GSE was mostly located in the left anterior thalamic radiation. Thus, although these correlations did not survive correction for multiple comparisons, they nonetheless suggest that participants with higher increase of FA and decrease of RD have the higher increase of originality and GSE scores at T1. No significant correlations were found between longitudinal changes of diffusion parameters and AUs’ fluency and flexibility subscales scores, as well as motivation scores.

**Table 6:** Results of voxelwise correlation analyses between longitudinal changes in FA and RD maps and concomitant changes in behavioral tests (*p* < 0.005 uncorrected).

			MNI coordinates			
Cluster size	*T*	*p*	*x*	*y*	*z*	WM structures
**FA - Originality ↑**						
34	2.65	<0.001	15	–2	7	Right anterior thalamic radiation/ *Right Posterior limb of internal capsule*
23	3.31	<0.001	–34	–18	35	Left superior longitudinal fasciculus
**RD – Originality ↓**						
56	3.44	<0.001	–17	–14	55	Left superior longitudinal fasciculus
38	4.26	<0.001	16	–2	6	*Right posterior limb of internal capsule*
31	3.31	0.001	–29	–3	27	Left superior longitudinal fasciculus (temporal part)
**FA – GSE ↑**						
82	3.33	<0.001	–23	18	13	Left anterior thalamic radiation
30	4.31	<0.001	–22	–3	15	Left anterior thalamic radiation / *Left Anterior limb of internal capsule*
29	3.88	0.001	9	–7	11	Right anterior thalamic radiation
24	4.79	<0.001	–37	–24	31	Left superior longitudinal fasciculus, Left Superior longitudinal fasciculus (temporal part)
**RD – GSE ↓**						
29	3.51	0.001	–21	–3	16	Left anterior thalamic radiation/*Left anterior limb of internal capsule*
22	3.93	<0.001	–22	15	12	Left anterior thalamic radiation/Left *anterior limb of internal capsule*

*Positive correlations were found between regions showing longitudinal increments of FA and both AUs originality and GSE scores at T1 respect to T0. Negative correlations were also found between longitudinal decrements of RD and improvements of originality and GSE scores at T1 respect to T0. Refer to **Table 2** for a detailed explanation of the table layout.*

## Discussion

In this work, we investigated for the first time the longitudinal effects of daily QMT on WM microstructure in a healthy group of subjects. Of note, respect to conventional pre–post training longitudinal studies, our subjects were tested three times over a period of 12 weeks of QMT, to examine the trend of training-related microstructural WM changes over time. Furthermore, we used an unbiased DTI analysis pipeline for tracking longitudinal WM changes, following recent advances in tensor-based image registration (Zhang et al., 2006; Keihaninejad et al., 2013). We also investigated the possible relationships between longitudinal microstructural changes and concomitant changes in several well-being related measures of creativity, perceived self-efficacy and motivation.

Our results revealed that QMT daily practice significantly affected WM microstructural architecture over time. Respect to the baseline (T0), FA values increased after 6 weeks of training (T1) in different bilateral tracts and in major associative tracts of the left hemisphere. Significant training-induced FA increments were still present after 12 weeks of QMT (T2) respect to T0, although less widespread and only localized in the left-hemisphere. No significant FA changes were found at T2 respect to T1.

We also examined the pattern of AD and RD changes in tracts where FA significantly increased and found a significant decrease of RD both at T1 and at T2, supporting the relevance of myelination processes in training-related FA changes. Behavioral analyses showed that our subjects remained motivated during the entire course of training, confirmed and deepened the knowledge of the longitudinal effect of QMT on creativity and revealed a training-related effect on self-efficacy. Finally, we found significant correlations between individual WM microstructural changes and individual improvements of self-efficacy and originality. These findings support the effectiveness of QMT in improving WM integrity and suggest the relevance of these microstructural changes for psychological well-being.

### QMT-Induced Longitudinal Effect on WM Microstructure

The unique combination of motor and cognitive components, which distinguishes the QMT from other mindfulness practices, could explain the present pattern of results, which comprises WM tracts related to sensorimotor functions as well as critical tracts for high-level cognitive operations.

Respect to T0, significant FA increments were found bilaterally in the corticospinal tract at T1. These changes are probably related to the sensorimotor effect of the QMT, in accordance with previous studies (Bengtsson et al., 2005; Wang et al., 2014). However, these FA increments only persisted in the left hemisphere at T2. This asymmetry could be attributed to handedness; since all participants were right-handers, the left corticospinal tract could be more susceptible to the long-term sensorimotor effect of training than the right tract (Imfeld et al., 2009). Furthermore, several studies reported a left hemisphere specialization for precise control of motor actions on both sides of the body in right-handers (for a review see Sadeghi et al., 2000), which extends to motor learning (Schambra et al., 2011). With practice, motor associated areas of the left-hemisphere reveal increased activity, also suggesting a left-hemispheric dominance in the storage of visuomotor skills (Halsband and Lange, 2006). We also observed an increase of FA in the body of corpus callosum at T1, suggesting an increase of interhemispheric communication between motor areas taking place in the first phase of training. It cannot be excluded that such changes could reflect cross-hemispheric transcallosal inhibition processes (Fling et al., 2013), which might contribute to the left-lateralized FA increase of the corticospinal tract at T2.

Significant FA increments were also found at T1 in the anterior thalamic radiations, which are generally related to executive function, memory encoding and planning of complex behaviors (Van der Werf et al., 2003; Mamah et al., 2010). Executive functions and memory are central to plan and perform movements in the correct sequence as well as to navigate in the Quadrato space. The FA increments only survived in the left hemisphere at T2 respect to T0. Longitudinal DTI studies have already reported significant FA increments in the left anterior thalamic radiation after memory training (Engvig et al., 2012; de Lange et al., 2016). Furthermore, this fiber tract has been recently related to gait stability and speed (Bruijn et al., 2014; Vercruysse et al., 2015), both crucial aspects for the correct execution of the QMT.

Other significant microstructural FA increments were found at T1 in both left and right uncinate fasciculi, which persisted in the left-hemisphere at T2. These fasciculi play a role in emotion regulation, emotional learning, memory, and language functions (Papagno et al., 2011; Von Der Heide et al., 2013; Holzel et al., 2016). Microstructural changes in these fiber bundles were somewhat expected, since QMT already proved its validity in improving emotional well-being (Ben-Soussan, 2014), emotional regulation (Ben-Soussan et al., 2017), and reading performance (Ben-Soussan et al., 2014a).

Fractional anisotropy increments at T1 were also localized in the genu of the corpus callosum and forceps minor, as well as in the superior cerebellar peduncles. Longitudinal increases of FA in the genu and forceps minor have been already reported after mindfulness (Tang et al., 2010; Tang et al., 2012), as well as memory trainings (Salminen et al., 2016). The forceps minor connects, via the genu, the prefrontal cortices (Hofer and Frahm, 2006; Park et al., 2008). Interestingly, this interhemispheric communication it is thought to be involved in locomotion (Wang et al., 2012; Bolandzadeh et al., 2014), and in lower extremity control (Fling et al., 2016). Furthermore, the prefrontal cortex forms close connections with the cerebellum, connected to the midbrain by the superior cerebellar peduncles (Allen et al., 2005; Krienen and Buckner, 2009; Watson et al., 2014). Prefrontal cortex and cerebellum are both engaged when high level of attention and concentration are required, especially during challenging and novel tasks (Diamond, 2000). Therefore, the present FA changes could be related to the higher levels of sensorimotor coordination, balance and attention required during the first weeks of QMT.

At T1, an increase of FA was also detected in the left superior longitudinal fasciculus, which connects parietal to frontal ipsilateral regions, as well as in its temporal part, which instead connects temporal with ipsilateral frontal areas, also including fibers belonging to the arcuate fasciculus (Mori and Crain, 2005; Wakana et al., 2007; Madhavan et al., 2014). Microstructural WM integrity of both superior longitudinal and arcuate fasciculi in the left hemisphere has been associated with auditory and verbal working memory as well as several language functions (Peters et al., 2012; Lopez-Barroso et al., 2013; Yeatman and Feldman, 2013; Urger et al., 2015; Piervincenzi et al., 2016). Moreover, recent studies have reported a strong relationship between visuospatial and attentional processing and this fasciculus (Thiebaut de Schotten et al., 2011; Bartolomeo et al., 2012; Chechlacz et al., 2015). The QMT requires a state of enhanced attention: the subject needs to pay attention to the verbal instruction (auditory working memory) but at the same time focus on his/her higher-order body location to remain in the Quadrato space. The increased connectivity between auditory and motor areas [connected by the arcuate fasciculus (Catani et al., 2005)] and between parietal body awareness and attention areas and prefrontal executive regions [connected by the superior longitudinal fasciculus (Thiebaut de Schotten et al., 2011)] could be explained by the high attentional demand of the QMT.

Robust long lasting (at both T1 and T2 respect to T0) left-lateralized FA increases were present in two long association fiber tracts, the inferior longitudinal and inferior fronto-occipital fasciculi. While the inferior longitudinal fasciculus is thought to mediate fast transfer of visual signal to temporal regions (Catani et al., 2003), and to play an important role in visual recent memory and language (Ffytche and Catani, 2005; Mandonnet et al., 2007; Ross, 2008), the functions of the inferior fronto-occipital fasciculus are not clearly understood. However, it has been suggested that it participates to reading, semantic processing, attention, and visual processing (Catani and Thiebaut de Schotten, 2008; Almairac et al., 2015). The attentional and working memory visuospatial resources required for the correct execution of the QMT are likely to contribute to the microstructural changes in these fiber tracts. However, since they have been also related to language functions in the left hemisphere, effects of QMT on language abilities should be also taken into consideration. As a matter of fact, this practice have already proved its utility in improving reading performance in dyslexic populations (Ben-Soussan et al., 2014a), which showed decreased WM integrity in language-related pathways (Steinbrink et al., 2008; Richards et al., 2015).

In the present work, no significant FA changes were instead found at T2 respect to T1. However, the repeated measures ANOVA showed that mean FA values at T2 were lower than at T1. This leads to the suggestion that the training-related increase of FA could have already met at T2 a descending phase. Notably, the same trend has been previously reported in several domains of expertise, such as motor sequence learning (Doyon et al., 2002), language acquisition (Sakai, 2005) and concentration meditation (Brefczynski-Lewis et al., 2007). This trend may be related to an adaptation component, as well as to offline processes, such as skill stabilization and improvement, which reflect memory consolidation (Dayan and Cohen, 2011). Long-term follow-up studies are required to further elucidate the present findings and confirm/disconfirm persistent or cumulative effects of QMT practice on WM microstructure.

### QMT-Related Left-Lateralized Microstructural Changes

Although previous studies reported left-lateralized changes following different types of training like working memory (Takeuchi et al., 2010; Salminen et al., 2016) or reading training (Keller and Just, 2009), little is known about the lateralization of WM microstructural changes related to mindfulness practices (Fox et al., 2014). Very few longitudinal studies investigated on the effects of mindfulness practices on WM integrity, with somewhat conflicting results about the lateralization of WM microstructural changes. Tang et al. (2010) reported left-lateralized FA changes in several fiber tracts, after 4 weeks integrative body-mind training (IBMT), a specific form of mindfulness meditation. In a second study, the same authors investigated IBMT-related longitudinal changes of FA, RD and AD in two separate ethnic groups of subjects performing 2 or 4 weeks of training, and reported similar left-lateralized WM changes (Tang et al., 2012). Conversely, Holzel et al. (2016) reported a specific FA increase in the right uncinate fasciculus after 8 weeks of Mindfulness-Based Stress Reduction (MBSR) course, which was also related to behavioral changes in emotional learning. Further research is needed to better address the link between WM hemispheric asymmetries and mindfulness practices, also using dedicated methods for the quantitative assessment of WM hemispheric lateralization (Catani et al., 2007; Perlaki et al., 2013).

### QMT-Related Changes as a Result of Myelination Process

Likewise the present results, several works reported the same pattern of FA increase and RD decrease after training (Keller and Just, 2009; Hu et al., 2011; Engvig et al., 2012). Since RD is thought to reflect the myelination degree (Fields, 2010; Hu et al., 2011), these studies supported the idea that myelination could be the leading process of the increased FA following training. Myelination has been found, in both animal and humans, to be modifiable by experience (Ishibashi et al., 2006; Fields, 2008, 2010). It has been proposed that training would increase neural firing and thus increase myelination, that enhances communication among cortical areas and may result in better performance (Keller and Just, 2009). Evidence on active myelination process after 12-week QMT training has been already described by Ben-Soussan et al. (2015c), who reported increased levels of proBDNF, which directly affects myelination by regulating the development of oligodendrocyte progenitor cells (for a review see also Zatorre et al., 2012). Therefore, the present results could support QMT as an effective myelination-promoting intervention.

### Correlations between QMT-Related WM and Behavioral Changes

In the present work, training-induced improvements in originality and general self-efficacy (GSE) were associated with increased FA/decreased RD in the right anterior thalamic radiation, and left superior longitudinal fasciculus. Creativity measures have been previously related to the anterior thalamic radiation, a fiber tract associated to creative cognition (Jung et al., 2010). Little instead is known about possible relationships between the superior longitudinal fasciculus and creativity, although it has been suggested a role of this tract in the process of creative thinking (Fink and Neubauer, 2006).

Similar to the originality results, we found that higher GSE scores were associated with increased FA/decreased RD in the anterior thalamic radiations and left superior longitudinal fasciculus. This is the first study that investigated on possible correlations between training-related WM changes and measures of self-efficacy. GSE has been positively related to optimism, self-respect and internal control (Bandura, 1997; Magaletta and Oliver, 1999) and negatively associated with anxiety and depression (Endler et al., 2001; Tahmassian and Jalali Moghadam, 2011). Interestingly, anxious and depressed patients also showed alteration in the WM integrity of both superior longitudinal fasciculus and anterior thalamic radiation (Lai and Wu, 2014; Albaugh et al., 2016). Furthermore, a recent large-scale study (*n* = 776 healthy subjects) of Nakagawa et al. (2015) reported significant correlations between GSE scores and WM density of prefrontal, parietal regions and temporo-parietal junction, all regions interconnected by the superior longitudinal fasciculus, suggesting a pivotal role for these areas in self-cognition, self-efficacy and social cognition. The present correlation between GSE scores and QMT-related microstructural changes in the above-mentioned tracts strengthens previous claims that mindfulness practices can effectively improve self-efficacy and supports the idea that one mean of QMT to promote psychological well-being is by enhancing self-confidence and copying skills (McAuley and Blissmer, 2000; Cataldo et al., 2013; Sanaei et al., 2014).

### Limitations of the Study

There are a few limitations to this study, which should be noted. The first is the lack of a control group with no training or a control group with the same type of motor activity (but with reduced cognitive demands) or cognitive effort (but reduced motor load). However, several studies have already demonstrated the longitudinal reliability of DTI measures, including previous learning studies where control groups did not show FA changes (Scholz et al., 2009; Taubert et al., 2010). Furthermore, our previous studies have already demonstrated that the neuronal and cognitive changes are QMT specific, compared to different control groups controlling separately for cognitive and motor load (Dotan Ben-Soussan et al., 2013; Ben-Soussan et al., 2014b; Venditti et al., 2015), thus providing more confidence in the findings of the present work. A second limitation is that we used DTI images of 12 diffusion directions with a number of scan repetitions of four. Although more scan repetition seem to be related to a higher signal-to-noise ratio and more reliable FA and tractography data (Jones, 2004; Farrell et al., 2007), it has been suggested that a larger number of diffusion gradient directions may improve the estimation of the diffusion tensor (Jones, 2004; Ni et al., 2006). Thus, future controlled QMT studies should include more directions of the diffusion gradient.

## Conclusion

The effectiveness of the Quadrato Motor Training, together with the ease of learning and the minimal time and space it requires, make this training a very promising and feasible paradigm for children, adolescents and elderly people, contributing to well-being in the healthy but also useful for neurorehabilitation. Future research should examine QMT efficacy on different populations suffering from altered WM microstructural connectivity and impaired cognitive performance, such as mild cognitive impairment patients, or with decreased motor and/or cognitive functions, such as learning disabilities, language disorder and Parkinson disease. Furthermore, exploring how WM microstructural changes are related to measures of well-being and health may have relevant implications for cognitive and educational neuroscience as well as psychotherapeutic programs.

## Author Contributions

TB-S, FC, and CQ designed the research. CQ, CM, and YE performed the research. CP, FC, and FM analyzed the data. CP and FC wrote the paper. TB-S, FM, and CQ contributed to the writing process.

## Conflict of Interest Statement

The authors declare that the research was conducted in the absence of any commercial or financial relationships that could be construed as a potential conflict of interest.

## Footnotes

1 http://www.fmrib.ox.ac.uk/fsl/

2 http://dti-tk.sourceforge.net/pmwiki/pmwiki.php

3 http://www.fmrib.ox.ac.uk/fsl/fslwiki/Atlases
